# Efficacy of auricular plaster therapy for sleep disorders in preschool children with autism spectrum disorders: Study protocol for a randomized controlled trial

**DOI:** 10.3389/fneur.2022.973609

**Published:** 2022-10-03

**Authors:** Duoxi Duan, Lin He, Hong Chen, Ying Lei, Wei Wu, Tao Li

**Affiliations:** ^1^Department of Integrated Traditional Chinese and Western Medicine, The Second Affiliated Hospital of Chengdu Medical College, China National Nuclear Corporation 416 Hospital, Chengdu, China; ^2^Foreign Language School, Southwest Medical University, Luzhou, China; ^3^Department of Anesthesiology, The Second Affiliated Hospital of Chengdu Medical College, China National Nuclear Corporation 416 Hospital, Chengdu, China

**Keywords:** auricular plaster therapy, sleep disorders, autism, preschool children, study protocol

## Abstract

**Background:**

Children with autism spectrum disorders (ASDs) suffer from sleep disorders to a considerable degree; however, there is no safe and effective treatment available in clinical practice. The objective of the trial is to assess the clinical effectiveness of auricular plaster therapy (APT) in treating sleep disorders in children with ASD.

**Method:**

This is a single-center, patient-assessor blind, randomized controlled trial. A total of 44 preschool children with sleep disorders with ASD will be included in this study. Eligible participants will be randomly assigned to either the auricular plaster group or the sham auricular plaster group in a 1:1 ratio. Participants in the different groups will receive APT or sham APT, respectively, for a total of 30 sessions over 30 days. The primary outcome includes the Children's Sleep Habits Questionnaire (CSHQ), while secondary outcomes include the Autism Behavior Checklist (ABC) and polysomnography (PSG) for total sleep time, sleep latency, awakening duration, and sleep structures. The CSHQ and ABC will be assessed at baseline, 10, 20, 30, 60, 90, and 120 days after randomization, whereas PSG will be assessed at baseline and 30 days after randomization. The follow-up period will be scheduled to be 60, 90, and 120 days after randomization.

**Discussion:**

The results of this study may provide evidence of the efficacy of APT, as well as offer new alternatives for the treatment of sleep disorders in children with ASD.

**Trial registration:**

CHiCTR.org.cn (ChiCTR2100048257). Registered on July 5, 2021.

## Introduction

Autism spectrum disorder (ASD) is a complex developmental condition characterized by difficulties in social interaction, communication, and common repetitive behavior patterns (1). Globally, the World Health Organization reported that 0.76% of children have ASD, accounting for 16% of the total number of children throughout the world (2). It is estimated that over 44% of children with ASD will develop sleep disorders, and these sleep disorders exist for a long period of time (3, 4). Sleep disorders commonly present as difficulty falling asleep, poor sleep quality, wakefulness, irregular sleep patterns, short sleep duration, and a tendency to wake up during the night (5).

As outlined by published guidelines (6–10) (Table 1), the primary treatment for autistic children with sleep disorders is to improve sleep habits. For parents of children with ASD, it will take a long time and a great deal of patience to teach children proper sleep habits, such as a comfortable sleep environment, regular bedtimes, encouraging the child to sleep alone, and avoiding naps (11). Moreover, it is unclear whether improving sleep habits could effectively treat sleep disorders in children with ASD (12).

**Table 1:** Summary of clinical guidelines for the treatment of sleep disorders in children with autism spectrum disorders.

**Guidelines**	**Recommendations of treatment for sleep disorders**
2021 NICE guideline	Not mentioned
2020 AAN guideline	Behavioral strategies are first-line treatment approach (Level B). Clinicians should offer melatonin to children and adolescents with ASD if behavioral strategies have not been helpful (Level B). No evidence to support the routine use of weighted blankets or specialized mattress technology for improving disrupted sleep (Level B).
2017 BAP guideline	Melatonin, if possible, in combination with a behavioral intervention (Strength of recommendation: A). Prolonged use of benzodiazepines and related GABA agonists is not recommended (Strength of recommendation: S).
2017 NICE guideline	Not mentioned
2013 NICE guideline	Not mentioned

NICE, National Institute for Health and Care Excellence; ASD, autism spectrum disorders; AAN, American Academy of Neurology; BAP, British Association for Psychopharmacology; GABA, gamma-aminobutyric acid.

After reviewing currently published randomized controlled trials (RCTs) for sleep disorders in children with ASD, we found that these studies had several limitations: relatively small sample sizes (<40 participants), a less rigorous study design (unblinding method), and adopted different diagnostic criteria for ASD (12–25) (Table 2). The only drug currently available in children with ASD is melatonin, which regulates circadian rhythms and improves sleep (26). The first RCT on melatonin, with 11 participants, showed benefits for sleep disorders in children with ASD (25). Another trial concluded that melatonin had efficacy for sleep disorders only for a short duration (22). In 2021, Hayashi et al. (13) conducted an RCT of melatonin in 196 children and reported that melatonin was effective in treating sleep disorders in children with ASD. Nevertheless, melatonin was also associated with some adverse events (AEs), including nervous system disorders, infections and infestations, and pharyngitis. According to the 2020 American Academy of Neurology guideline, melatonin has not been clinically evaluated for safety, and its potential role in decreasing sleep disorders in children with ASD is dubious (11, 18). Moreover, taking melatonin has some potential side effects, such as enuresis, headache, and dizziness (11, 27). Other complementary alternative medicines to treat sleep disorders in children with ASD, such as aquatic exercise (14), ferrous sulfate (16), carnosine (17), weighted blanket (20), lack evidence-based recommendations, and thus remain controversial (11).

**Table 2:** Randomized controlled trials of sleep disorders in children with autism spectrum disorders.

**References**	**Country**	**Center**	**Intervention group**	**Control group**	**Sample size**	**Blinding**	**Primary outcomes**	**Conclusion**
Hayashi et al. (13)	Japan	Multicenter	Melatonin	Placebo	196	Double blind	Sleep onset latency	Melatonin is effective for sleep disorders.
Ansari et al. (14)	Iran	Single	Aquatic exercise	None	40	Not mentioned	CSHQ	Aquatic exercise may improve sleep quality and reduce the serum IL-1β and TNF-α.
Papadopoulos et al. (15)	Australia	Single	Sleep behavioral intervention	Usual clinical care	61	Not mentioned	CSHQ	A brief behavioral sleep intervention can improve sleep problems.
Reynolds et al. (16)	USA	Single	Ferrous sulfate	Placebo	20	Double blind	Bedtime and wake time	No improvement in insomnia in treated with ferrous sulfate.
Mehrazad-Saber et al. (17)	Iran	Single	Carnosine	Placebo	43	Double blind	CSHQ	Carnosine could be effective in improving sleep disorders.
Gringras et al. (18)	USA	Single	PedPRM	Placebo	125	Double blind	SND and CSDI	PedPRM is effective and safe for treatment of insomnia.
Frazier et al. (19)	USA	Single	Pre-STS mattress	After-STS mattress	45	Double blind	Sleep diary	STS could improve sleep duration and sleep efficiency.
Gringras et al. (20)	USA	Single	Weighted blankets	Placebo	73	Not mentioned	TST	The use of a weighted blanket does not help children with ASD sleep.
Johnson et al. (21)	USA	Single	BPT program for parents	Not BPT	40	Not mentioned	Treatment fidelity checklist	BPT has a certain effect on sleep disorders.
Cortesi et al. (22)	Italy	Single	CBT and melatonin	Melatonin or Placebo	160	Double blind	Sleep variables*	In the short term, CBT and melatonin have efficacy for sleep disorders
Adkins et al. (12)	USA	Single	Sleep education to parents	No sleep education	36	Not mentioned	Changes in sleep latency	The sleep education pamphlet did not improve sleep latency.
Wright et al. (23)	UK	Single	Melatonin	Placebo	22	Double blind	Sleep variables^†^	Melatonin improved sleep latency and total sleep but not number of night awakenings.
Wirojanan et al. (24)	USA	Single	Melatonin	Placebo	12	Double blind	Sleep variables^‡^	The efficacy and tolerability of melatonin treatment for sleep problems can be affirmed.
Garstang and Wallis (25)	UK	Single	Melatonin	Placebo	11	Double blind	Sleep variables¶	Melatonin was beneficial for sleep disorders.

CSHQ, children's sleep habits questionnaire; TNF, tumor necrosis factor; PedPRM, prolonged-release melatonin minitablets; SND, sleep and nap diary; CSDI, composite sleep disturbance index; STS, sound-to-sleep; TST, total sleep time; BPT, behavioral parent training; CBT, cognitive behavioral therapy.

^*^Represents sleep latency, total sleep time, wake after sleep onset, and the number of awakenings in sleep variables.

^†^Represents sleep latency, total sleep, and night awakening in sleep variables.

^‡^Represents sleep-onset time, total night sleep duration, sleep-onset latency time, and the number of night awakenings in sleep variables.

¶Represents sleep latency, number of awakenings, and total sleep duration in sleep variables.

As a part of traditional Chinese medicine (TCM), acupuncture is a vital component with a long history of treating diseases such as mental illness (28–30), cardiovascular disease and cerebrovascular disease (31), and tumor disease (32, 33). Auricular therapy is one treatment modality of acupuncture, which involves stimulating specific acupoints on the outer ear in an effort to promote health and wellbeing (34). As a form of auricular therapy, auricular plaster therapy (APT) is composed of a round and hard cowherb seed and a sticky adhesive tape with a size of 0.5 cm ^*^ 0.5 cm (35). Due to the non-invasive, safe, and convenient nature, once auricular plaster is affixed by the doctor, patients themselves can press and stimulate the points at the convenience of their own time. Although the mechanism by which APT treats insomnia is not fully understood, numerous studies have indicated that APT helps relieve insomnia by modulating neurotransmitter activity and affecting the nervous system (36). Several systematic reviews and meta-analyses of APT show that APT appears to be an effective and safe treatment for patients with primary insomnia (37–41). In the most recent study, a retrospective cohort study of APT treatment of 84 patients with coronavirus disease 2019 (COVID-19) with insomnia showed that APT was effective in alleviating insomnia and anxiety (42). Another RCT of 50 patients receiving methadone maintenance treatment (MTT) showed that APT combined with electroacupuncture could significantly improve sleep quality, sleep latency, and increase MMT adherence (43). In addition, the application of APT has also been extended to the treatment of pain management (44, 45), postoperative rehabilitation (46), attention deficit (47), primary dysmenorrhea (48), and other conditions. Nevertheless, to our knowledge, there have been no RCTs evaluating the efficacy of APT for sleep disorders in children with ASD. Given that APT is effective in treating insomnia in adults, we aim to test the safety and efficacy of auricular plaster therapy for the treatment of sleep disorders in preschool children with ASD, which may provide a viable alternative treatment method.

## Methods and analysis

### Study design

This study is a parallel-design, patient-assessor blind randomized controlled trial (RCT) comparing the use of APT with sham APT. The recruitment of participants will take place from 1 August 2021 to 31 December 2022. The program will enroll autistic preschoolers with sleep disorders who will be assessed at the Sichuan Beidouxing Rehabilitation Service Center as well as three other community hospitals (Fuqin Community Health Service Center, Tiaodenghe Community Health Service Center, and Xianqiao Community Health Service Center). The clinical trial was registered on CHiCTR.org.cn (ChiCTR2100048257) before we enrolled our first participant, and the study was approved by the Second Affiliated Hospital of Chengdu Medical College, China National Nuclear Corporation 416 Hospital Ethics Committee (KJ2021012).

**Figure 1:**
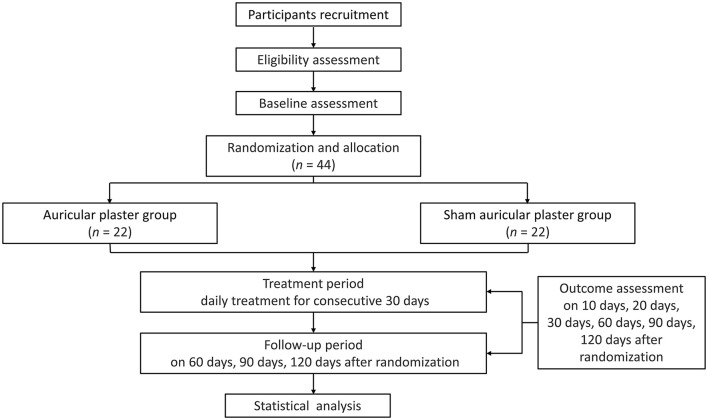
Study flowchart.

Eligible participants will be randomly assigned to the APT group or the sham APT group on a 1:1 basis. An observation period of 120 days will be conducted, including a 30-day treatment period and a 90-day follow-up period. Children will receive APT continuously for 1 month. Assessments will be conducted at baseline as well as 10, 20, 30, 60, 90, and 120 days after randomization. The study flowchart is shown in Figure 1, and the schedule of the trial is shown in Table 3.

**Table 3:** Study schedule of the trial.

	**Study period**							
	**Enrollment**	**Allocation**	**Post-allocation**				**Follow-up period**		
**Timepoint (days)**	**-7**	**0**	**1**	**10**	**20**	**30**	**60**	**90**	**120**
**Enrollment**									
Eligibility screen	**X**								
Informed consent	**X**								
Allocation		**X**							
**Interventions**									
Auricular plaster			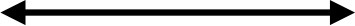						
Sham auricular plaster			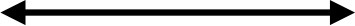						
**Assessments**									
CSHQ			**X**	**X**	**X**	**X**	**X**	**X**	**X**
ABC			**X**	**X**	**X**	**X**	**X**	**X**	**X**
PSG			**X**			**X**			
AEs				**X**	**X**	**X**			

CSHQ, Children's Sleep Habits Questionnaire; ABC, Autism Behavior Checklist; PSG, polysomnography; AES, adverse events.

The protocol complies with the Standard Protocol Items: Recommendations for Intervention Trials (SPIRIT) guidelines (49). The Consolidated Standards of Reporting Trials (50) as well as the Standards for Reporting Interventions in Clinical Trials of Acupuncture (51) provide a framework for designing this clinical trial.

### Participants

We will include preschoolers who meet the diagnostic criteria for autism and sleep disorders. Upon meeting the inclusion criteria and not meeting any of the exclusion criteria, an ASD child will be considered eligible.

#### Inclusion criteria

Participants who meet all of the following inclusion criteria will be included: (1) meet the diagnostic criteria for sleep disorders (ICSD-3) (52) and autism (1); (2) aged 2–6 years old; (3) did not receive relevant treatment measures 1 week prior to enrollment; and (4) written informed consent obtained from parents.

#### Exclusion criteria

Participants who meet any of the following criteria will be excluded: (1) known severe cardiovascular, cerebrovascular, liver, kidney, blood, and other systemic diseases; (2) known history of Asperger's syndrome, Heller syndrome, Rett syndrome, specific receptive language disorder, or childhood schizophrenia before (53–55); (3) known taking part in other clinical trials.

#### Drop-out criteria

Participants drop out for the following reasons: (1) they experience severe adverse events (SAEs) and are ineligible for further study; (2) they withdraw from the clinical study; and (3) they have manifestations of allergies to auricular plaster.

### Randomization and blinding

Based on a random number generated by SAS (Version 9.3, SAS Institute Inc., Cary, NC, USA), participants will be randomized in a 1:1 ratio to either the auricular plaster group or the sham auricular plaster group. Random numbers are generated by a statistician who is not participating in the trial. The random grouping results are sent to the acupuncturists by message. However, the particular characteristics of auricular plaster therapy make it difficult for acupuncturists to be blinded. Participants do not know which group they belong to. APT will be administered to participants in separate rooms according to their assigned groups. Researchers and statisticians in the trial will be blinded to the grouping scenario.

### Basic treatment regimen

Participants will receive standard rehabilitation training for autism according to the published guidelines (6), including physical activities, rhythm classes, sensory integration classes, discrete unit teaching methods, and natural environment teaching. These rehabilitations will be guided by qualified professionals.

### Interventions

**Figure 2:**
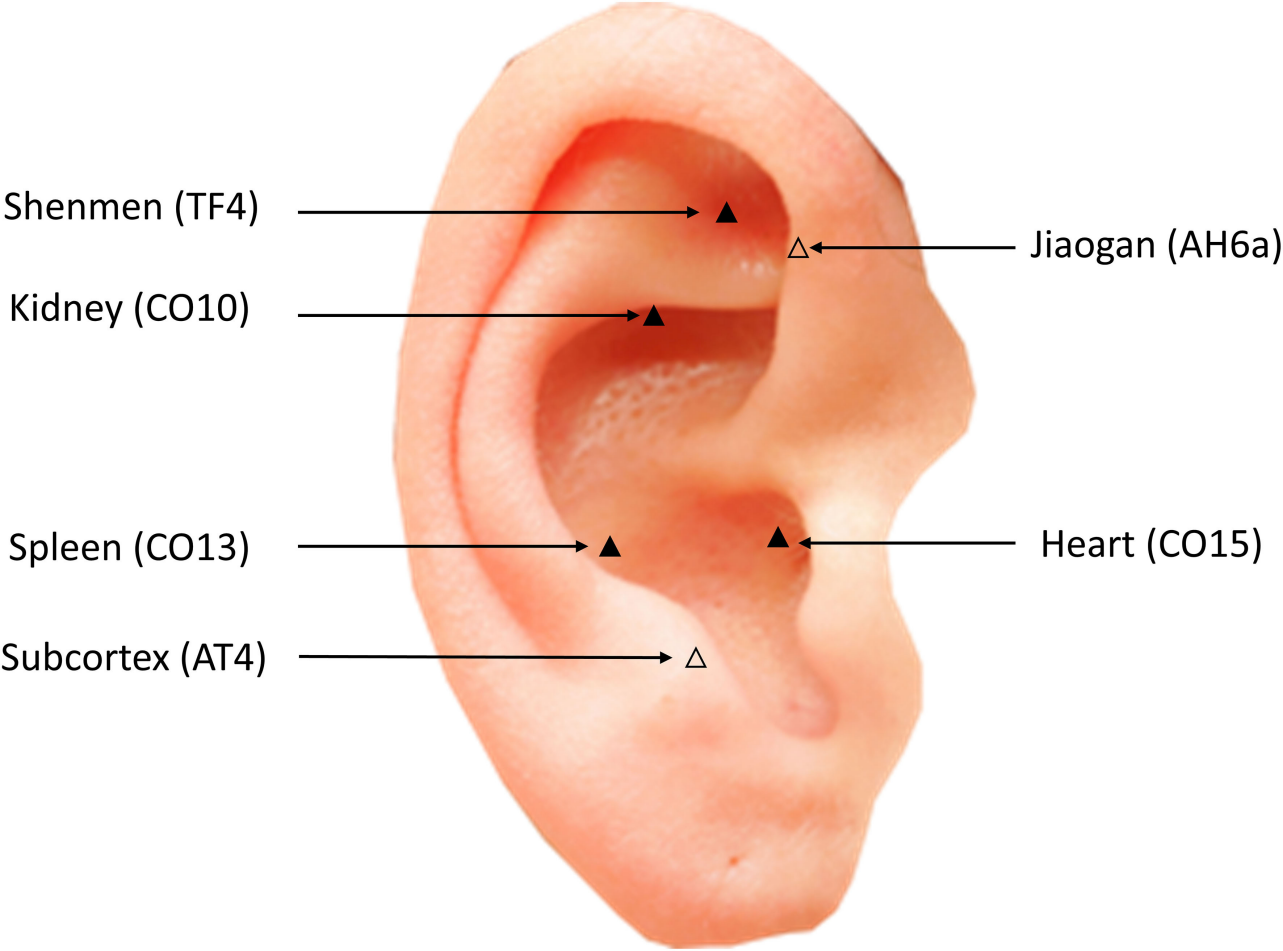
Location of auricular acupoints.

Based upon clinical experience in APT for sleep disorders and characteristics of TCM treatment of sleep disorders in children with ASD, the acupoints of the heart (CO15), Jiaogan (AH6a), Shenmen (TF4), subcortex (AT4), kidney (CO10), and spleen (CO13) are chosen in this study (56). The locations of the auricular points can be found in Table 4 and Figure 2. Both groups will be treated with APT for 30 consecutive days. Before participating in the trial, the acupuncturists qualified as Chinese medicine practitioners have at least 3 years of clinical experience, and successfully passed a relevant test, including auricular point positioning, auricular point duration, and participant attention. The auricular plaster (Heshi MedTech Co., Ltd., Hengshui, Hebei, China) consists of cowherb seeds wrapped in a tape of 0.5 cm ^*^ 0.5 cm. Participants in the sham APT group will only be treated with the same shape as auricular plaster without cowherb seeds. To increase compliance, we also manufacture special auricular plaster, which is covered with cartoon stickers. Acupuncturists will change the auricular plaster application for participants every day. Participants are not allowed to take any therapeutic drugs (e.g., melatonin) for sleep disorders during the study.

**Table 4:** Locations of auricular points.

**Auricular point**	**Location**
Heart (CO15)	Located at the middle of the concha
Jiaogan (AH6a)	Located at the junction of the front end of the lower part of the antihelix and the inner edge of the helix
Shenmen (TF4)	Located at the upper of the posterior third of the triangular fossa
Subcortex (AT4)	Located at the medial side of the antitragus
Kidney (CO10)	Located at the rear of the lower part of the antihelix
Spleen (CO13)	Located at the posterior upper part of the concha

#### Auricular plaster group

Based on conventional rehabilitation training for children with ASD, children in the auricular plaster group will be provided with APT. Children themselves or their parents are told to press the auricular plaster three times a day (8 a.m., 2 p.m., half an hour before sleep at night), each point for 30–60 s, so that the auricular point produces an acidic and swollen sensation that can be tolerated by the children.

#### Sham auricular plaster group

Participants will receive a sham APT on the same auricular point location as the APT group. They will not be instructed to press the sham plasters.

### Outcome measurements

#### Primary outcome

The primary outcome is the Children's Sleep Habits Questionnaire (CSHQ) (57), which contains seven items, including bedtime, sleep habits, sleep behavior, night wake, morning wake, daytime sleepiness, and total sleep time, with different items representing different sleep problems. Higher scores indicate a greater problem with sleep (58). The CSHQ will be evaluated at baseline and 10, 20, 30, 60, 90, and 120 days after randomization. Sleep assessment ranges from 1 month prior to baseline, every 10 days in the treatment period, and every 30 days in the follow-up period.

#### Secondary outcomes

Secondary outcomes include the following:

1) The polysomnography (PSG) system (Natus Neurology Incorporated, Wisconsin, USA), a clinical measure for evaluating sleep conditions, is considered to be the “gold standard” for determining sleep-related disorders (59). The sleep variables of PSG include the following: total sleep time (TST), sleep latency (SL), awakening duration, and sleep stages (non-rapid eye movement sleep stage 1 (NREM1), NREM2, NREM3, rapid eye movement (REM) sleep latency and REM sleep) (60). Sleep stages are determined by analyzing the electroencephalogram (EEG), electrooculogram (EOG), and chin electromyogram (EMG) recording. EEG derivations F4-M1, C4-M1, and O2-M1 are obtained from the electrical activity in frontal, central, and occipital brain regions. The frequency filter for these derivations ranges from 0.3 to 35 Hz. The EOG derivations E1-M2 and E2-M2 are determined by electrodes placed in the left and right outer canthus, respectively, with frequency filters ranging from 0.35 to 35 Hz. Three electrodes placed at the chin provide the EMG derivations EMG1, EMG2, and EMG3, which have a frequency filter of 10–100 Hz. Children with ASD will receive 16-h PSG monitoring at baseline and the end of treatment. The PSG data will be analyzed by Natus ^®^ SleepWorks™ PSG software. A certified neurosurgeon with 8 years of experience (Dr. Yan Ni) who has passed the Chinese PSG technical operation examination will audit the PSG data.
2) Autism Behavior Checklist (ABC), which is one of the five components of the Autism Screening Instrument for Educational Planning (61). There are 57 items divided into five parts in the ABC, which are categorized into five areas: sensory, relating, body and object use, language, and social and self-help skills. The ABC follows the same assessment schedule as the CSQH. The CSHQ and ABC questionnaires were assessed by one independent assessor who was blinded to group allocation.

### Sample size

A multisite case-control study which including 552 sleep problem in two to five children with ASD showed that the mean score of the CSHQ for ASD children was 48.5, and the standard deviation (SD) of CSHQ was 9.7 (62). Another study utilizing scalp acupuncture to treat sleep disorders in children with ASD showed a significant improvement in total CSHQ scores of 38 after treatment (63). Based on these two studies, we assume that the mean CSHQ score of children with ASD after receiving APT treatment is 40, and the SD in the auricular plaster group and sham auricular plaster group was 9.7. Assuming a significance level of 0.05 and a study power of 0.8 with a 10% dropout rate, 44 participants were required for this study, with 22 participants in each group. The sample size was calculated using power analysis and sample size (Version 11.0.7, NCSS, Englewood, New Jersey, USA).

### Data collection and management

Data of the participants will be stored in the case report forms (CRFs), and the data will be input into the electronic CRFs by a specialized data reader. Data are managed by the China National Nuclear Corporation Hospital Data Management Committee. Data will be checked by the manager once a month. Therapists will not have access to the data during the study.

### Quality control

All the researchers will be trained with the trial methodology and APT technique before the first participant is included. During the trial process, the China National Nuclear Corporation Hospital Data Management Committee is in charge of quality control.

### Adverse events and safety assessment

The AEs included allergies to auricular plaster, swelling, and severe pain. When SAEs occur that pose a threat to the participant's safety, the study will be stopped immediately and the blinding will be canceled to preserve the participant's life. A detailed record of all AEs/SAEs will be kept during the course of the study, including the date, duration, treatment measures, and results.

### Statistical analysis

An analysis of the data will be conducted using SPSS (Version 24, IBM, Armonk, New York, USA). Data will be analyzed on the basis of intention-to-treat (ITT) and per-protocol (PP) analysis. The ITT analysis includes all the participants who received at least one acupuncture treatment and one assessment of the primary outcome. The PP analysis includes participants who complete the trial. Continuous data will be expressed as medians and interquartile ranges. Categorical data will be presented as numbers and percentages. Continuous variables will be compared using the independent-sample *t-*test or the Mann–Whitney *U*-test. Categorical variables will be compared with the chi-squared test or Fisher's exact test. Missing values will be addressed by multiple imputations, having appropriately explored the missingness mechanism and in accordance with good practice. Two-sided *P* < 0.05 will be considered significant.

## Discussion

In children with ASD, sleep disorders are significantly more common than in normal children, which could lead to a lifelong problem if not addressed early on (4, 64). Sleep disorders are typically associated with communication difficulties and restrictive and repetitive behaviors, which are major symptoms of ASD. Children with ASD often suffer from sleep disorders, which adversely affect their moods, emotional regulation, behavior, and cognitive function. The consequences of abnormal behavior during the day can negatively impact the quality of sleep, resulting in a vicious cycle. Additionally, sleep disorders have a greater impact on obesity, injuries, and attention deficit in children with ASD than in other children (11, 65, 66).

Autism management is a lengthy and challenging process, which is a huge mental and economic burden on families. Children with ASD are often young and in their development stage. Treatments involving prescription drugs and complicated, painful, and invasive nonpharmacological therapies are not well accepted by children with ASD. In contrast to the placebo effect, acupuncture is one of the most effective ways to treat sleep disorders (67). Auriculotherapy is an important part of acupuncture (38), which can contribute to the improvement of sleep disorders for a variety of reasons (68–71). In this study, APT will be used to treat sleep disorders in children with ASD. It is a non-invasive, painless, and inexpensive treatment for children and is highly acceptable to both children and their parents. Hence, this acceptance could serve as a promising starting point for the study. In our study, parents are encouraged to participate and are taught how to press auricular plaster, which is in accordance with the guideline (6).

In recent years, there have been controversies regarding the effectiveness of both real acupuncture and sham acupuncture in treating disease. Sham acupuncture involves superficial needling and non-acupoint needling. A review of acupuncture for sleep disorders compared the efficacy of acupuncture, electroacupuncture, acupressure, and sham acupuncture/placebo (72), which showed that acupressure was more effective than sham acupuncture/placebo in improving sleep disorders.

As part of the study design, different groups of participants receive treatment in separate rooms, resulting in less communication between the groups and guaranteed blindness. Moreover, both the auricular plaster and sham auricular plaster have the same shape, which also prevents participants from identifying which group they belong to.

In TCM theory, ASD is attributed to a deficiency of the spleen and kidney. The main physiological functions of the spleen are to regulate transportation and transformation and dominate muscles and limbs (73). The function of the kidney is to store essence and maintain growth, development, and reproduction (73). By stimulating these two auricular points, children with sleep disorders and ASD can benefit from the improvement of their clinical symptoms by promoting musculoskeletal growth and transportation of qi and blood. Thus, the ear kidney (CO10) and ear spleen (CO13) were selected. As the heart regulates the blood vessels and governs the mind, TCM also believes that sleep disorders are closely related to the heart. *Inner Canon of the Yellow Emperor* states that “the heart is the residence of the spirit,” which means that good sleep is dependent on a sufficient supply of heart qi and enough blood. Shenmen (TF4) and heart (CO15) are most closely related to the heart and thus are selected. Moreover, the ear subcortex (AT4) can coordinate the excitatory and inhibitory functions of the cerebral cortex, and Jiaogan (AH6a) is able to regulate sympathetic nerve functions, which are closely related to the regulation of sleep. Thus, the acupoints of Jiaogan (AH6a) and subcortex (AT4) are selected in this study.

Outcome determination is of great importance for the trial. According to the pediatric International Classification of Sleep Disorders, the CSHQ is a classification scale designed specifically for diagnosing sleep disorders in school-aged children (57). As a scale for detecting sleep disorders in preschool children with abnormal sleep behaviors, the CSHQ has shown adequate reliability, validity, and internal consistency across long-term clinical studies (62, 74, 75), and in recent years, research has demonstrated that the CSHQ could be successfully applied to assessing sleep disorders in children with ASD (76–79). A study evaluating the psychometric properties of the CSHQ in 469 school-aged children (4–10 years old) with sleep disorders concluded that the CSHQ demonstrated internal consistency and test-retest reliability (57). Therefore, the CSHQ is used as the primary outcome to assess sleep disorders in children with ASD before and after treatment in this study. ABC is a well-established tool for screening and diagnosing autism (80, 81). Krug et al. (61) first investigated the psychometric properties of the ABC and found that the split-half reliability was 0.87. Subsequently, Yousefi et al. (82) assessed the psychometric features of ABC in 114 children (aged 6.82 ± 1.75) with ASD and found that the ABC can be used as an initial screening tool in the clinic. Thus, ABC is chosen as another outcome measurement. PSG can detect sleep problems that are often unnoticeable by other means, such as problems in sleep structure, sleep latency, and total sleep duration. In a cross-sectional study conducted by Aathira et al. (83) in 71 children with autism spectrum disorders, it was found that there was reduced sleep efficiency, decreased rapid eye movement, and reduced slow wave sleep duration in PSG, which may then affect the behavioral phenotype. Moreover, several studies also confirmed that children with ASD suffered from disrupted sleep structure, which included decreased REM sleep, longer sleep latency, lower sleep efficiency, and increased NREM1 sleep (84, 85). According to an RCT conducted in 2017, acupuncture could improve NREM1 and increase TST in patients with peri-menopausal insomnia (86). Therefore, PSG is selected as a secondary outcome to assess the effectiveness of APT in improving sleep structure.

Autistic preschool children with sleep disorders are recruited primarily from the Sichuan Beidouxing Rehabilitation Service Center and three other community hospitals. There are currently more than 200 preschool children with ASD in the Sichuan Beidouxing Rehabilitation Service Center, and approximately 50 preschoolers with ASD have enrolled in school annually, ensuring the inclusion of participants.

Our study has several limitations. First, the sample size of the trial is still relatively small, which is not a huge improvement compared with published studies. Second, researchers could not be blinded to group allocation because of the particularity of APT. Third, PSG is a challenge for children with ASD, although a specially designed PSG room was decorated in a cartoon style to improve adherence among children with autism and their parents accompany them at all times. Furthermore, PSG was monitored for only one night, resulting in an inevitable first-night effect.

In conclusion, the results of this study not only confirm the clinical efficacy of APT in treating sleep disorders in children with ASD but also provide new alternatives in the treatment of sleep disorders in children with ASD.

## Trial status

At the time of submission, recruitment of participants is currently underway.

## Ethics statement

The studies involving human participants were reviewed and approved by the Second Affiliated Hospital of Chengdu Medical College, China National Nuclear Corporation Hospital Ethics Committee (KJ2021012). Written informed consent to participate in this study was provided by the participants' legal guardian/next of kin.

## Author contributions

TL and DD contributed to the conception and design of this trial. DD, LH, and TL drafted the manuscript. HC planned randomization and statistical analysis. DD and YL participate in the recruitment and treatment of participants. WW is responsible for collecting the data. All authors contributed to the article and approved the submitted version.

## Funding

This study was supported by the Project of Sichuan Provincial Administration of Traditional Chinese Medicine (Grant No. 2021MS272).

## Conflict of interest

The authors declare that the research was conducted in the absence of any commercial or financial relationships that could be construed as a potential conflict of interest.

## Publisher's note

All claims expressed in this article are solely those of the authors and do not necessarily represent those of their affiliated organizations, or those of the publisher, the editors and the reviewers. Any product that may be evaluated in this article, or claim that may be made by its manufacturer, is not guaranteed or endorsed by the publisher.
